# Clinical Effect of Antioxidant Glasses Containing Extracts of Medicinal Plants in Patients with Dry Eye Disease: A Multi-Center, Prospective, Randomized, Double-Blind, Placebo-Controlled Trial

**DOI:** 10.1371/journal.pone.0139761

**Published:** 2015-10-12

**Authors:** Won Choi, Jae Chan Kim, Won Soo Kim, Han Jin Oh, Jee Myung Yang, Jee Bum Lee, Kyung Chul Yoon

**Affiliations:** 1 Department of Ophthalmology, Chonnam National University Medical School and Hospital, Gwangju, Korea; 2 Department of Ophthalmology, Chung-Ang University Hospital, Chung-Ang University College of Medicine, Seoul, Korea; 3 Department of Dermatology, Chonnam National University Medical School and Hospital, Gwangju, Korea; The Chinese University of Hong Kong, HONG KONG

## Abstract

**Purpose:**

To investigate the clinical efficacy and safety of wearable antioxidant glasses containing extracts of medicinal plants in patients with mild dry eye disease (DED).

**Methods:**

Fifty patients with mild DED were randomly assigned to wear either extracts of antioxidant medicinal plants containing (N = 25) or placebo glasses (N = 25). Patients wore the glasses for 15 min three times daily. The ocular surface disease index (OSDI) score, tear film break up time (BUT), and Schirmer’s test were evaluated and compared within the group and between the groups at baseline, 4 weeks, and 8 weeks after treatment.

**Results:**

OSDI score and tear film BUT were significantly improved in the treatment group at 4 and 8 weeks after wearing glasses (all *P* < 0.001). Compared to the placebo group, the OSDI scores were significantly lower in the treatment group at 8 weeks (*P* = 0.007). The results of the Schirmer’s test showed significant improvement in the treatment group at 4 weeks (*P* = 0.035), however there were no significant differences between the other groups or within the groups. No adverse events were reported during the study.

**Conclusions:**

Antioxidant glasses containing extracts of medicinal plants were effective in improving in DED both subjectively and objectively. Wearing antioxidants glasses might be a safe and adjunctive therapeutic option for DED.

**Trial Registration:**

ISRCTN registry 71217488

## Introduction

Dry eye disease (DED) is one of the most common ocular surface disorder that significantly affects quality of human life. Epidemiologic studies have reported that more than 15% of people suffer from DED worldwide. In the developed world, DED has become one of the leading reasons for patients to seek ophthalmological care.[1] Although the pathogenesis of DED has not been established clearly, it is well known that immune-mediated inflammation on the ocular surface plays a prominent role.[2] Current treatments for DED include life style modification, topical lubrication with artificial tears, topical anti-inflammatory and immune-modulatory agents, or punctal occlusion.[3] However, such therapeutic methods are often unsatisfactory or not well tolerated.

Recently, there is growing evidence that oxidative stress may be involved in cellular injury that leads to ocular surface diseases such as DED.[4] Oxidative stress generates reactive oxygen species (ROS) that can cause deleterious alterations in deoxyribonucleic acid, lipid, and protein of corneal and conjunctival epithelial cells. These damaged cells release cytokines that promote the ocular surface inflammation that results in DED.[5] Based on the role of oxidative stress, many antioxidants including sea buckthorn oil, green tea polyphenols, omega-3 essential fatty acids, and selenoprotein P have been demonstrated to reduce inflammation in corneal epithelial cells and improve tear film and ocular surface in experimental or clinical dry eye.[6–10]

Lee et al.[11] recently reported that thermal massage goggles were safe and effective for relieving symptoms and signs of DED. However, their treatment effects resulted from a thermodynamic mechanism, not regarding the core mechanisms of DED such as inflammation or oxidative stress.

Previously, we have demonstrated the efficacy of a mixture of ethyl alcohol (EtOH) extracts of medicinal plants, including *Schizonepeta tenuifolia var*. *japonica Kitagawa*, *Angelica dahurica Bentham ET hooker*, *Rehmannia glutinosa Liboschitz var*. *purpurea Makino*, and *Cassia tora L*. which have antioxidant and anti-inflammatory properties for protecting human corneal epithelial cells against oxidative stress induced by short wave length light emitting diode (LED) irradiation.[12] We hypothesized that local delivery of these antioxidant agents via glasses might be effective for controlling oxidative damages associated with DED. In the present study, we have evaluated the clinical efficacy and safety of the glasses containing extracts of various antioxidant medicinal plants in patients with mild DED.

## Materials and Methods

### Ethics statement

The study protocol, consent form, and data accumulation methods used in this were approved by the Institutional Review Board of Chonnam National University Hospital, Chung-ang University Hospital on September 28^th^, 2012 and the Korean Food and Drug Administration (No. MDCTC_2011_BM) on August 20^th^, 2012 and the study protocol followed the guidelines of the Declaration of Helsinki. The International Standard Randomized Controlled Trial Number (ISRCTN) is ISRCTN71217488. Registration before start of recruitment was unfortunately overseen by the investigators. The authors confirm that all ongoing and related trials for this treatment are registered.

### Study Population

This prospective, multicenter, double-blind, randomized, placebo-controlled clinical trial was conducted at two clinical research centers in South Korea from October to December 2012: Chonnam National University Hospital and Chung-Ang University Hospital. Written informed consent was obtained from all participants. The report of the study follows the CONSORT guidelines (S1 CONSORT Checklist). Study protocols are attached (S1 and S2 Protocols).

Patients aged 20 to 60 years with DED were recruited. The eligibility criteria for patients entering the study were based on the following: (1) one or more dry eye-related ocular symptoms (> 3 months) such as dryness, irritation, and burning sensations; (2) Ocular Surface Disease Index (OSDI) score of 13 to 32 (mild to moderate); and (3) tear film break-up time (BUT) of <10 s or Schirmer’s test (with application of local anesthetic) value <10 mm for 5 min.

Exclusion criteria were patients with pregnant woman, active eye and periocular skin inflammation, vitamin A deficiency, previous ocular surgery within 3 months before the study, a history of wearing contact lenses, a history of active treatment for dry eyes such as punctal occlusion or the usage of anti-inflammatory eye drops (topical steroid or topical cyclosporin) within 1 month of the study, and systemic condition or medication that could cause dry eye.

### Study design

**Fig 1:**
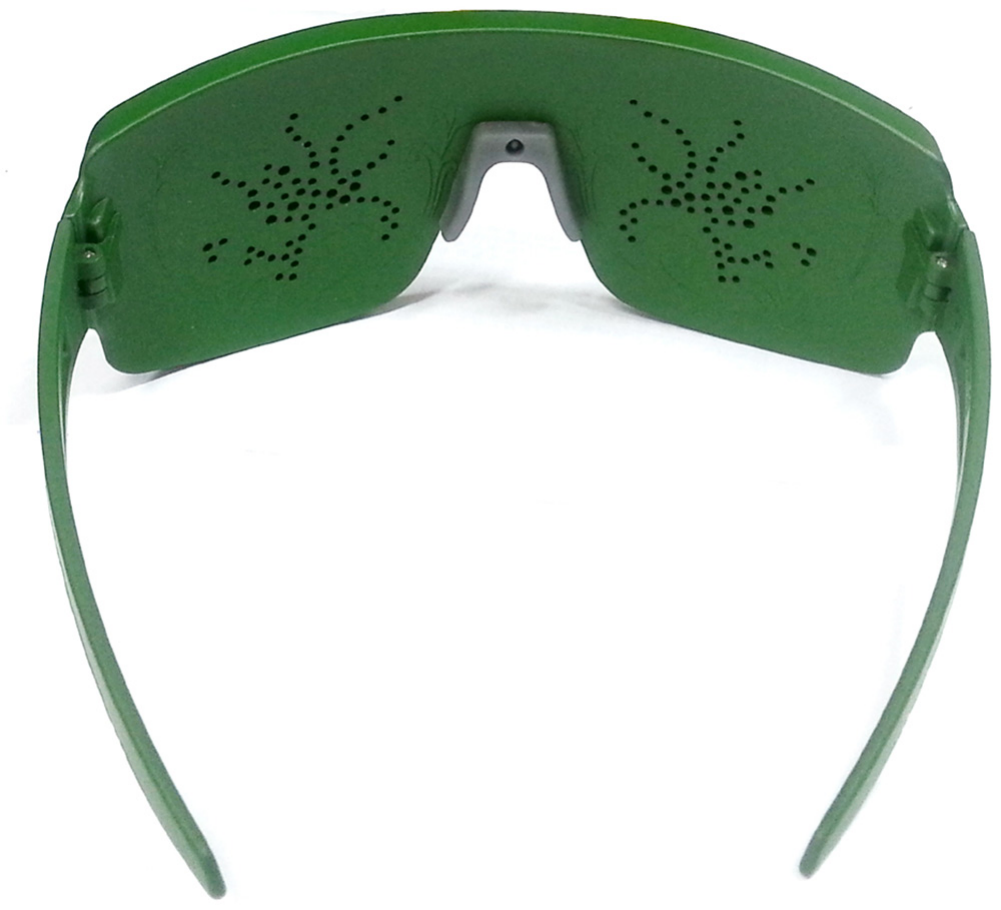
Mixed antioxidant medicinal plant extracts containing wearable glasses (EPA II-alpha, BM Biotechnology, Sunchon, South Korea).

Patients were recruited between October 1, 2012 and October 24, 2012. Patients were randomly assigned to either treatment or placebo groups. Participants of the treatment group received commercially available glasses (EPA II-alpha, BM Biotechnology, Sunchon, South Korea) (Fig 1). The glasses are composed of an external supporting frame with pads that contain mixed antioxidant medicinal plant extracts at the inside of frontal part of frame. There was a distance between eyelid and glasses. The participants opened their eyes as usual and there was no restriction of blinking during treatment sessions. The external supporting part of the frame is designed to wrap around the eye. Identical frames with pads that did not contain the medicinal plant extracts were provided to patients in the placebo group. Both treatment and placebo glasses were supplied in identical containers for masking purposes. The pad was composed of polypropylene and viscos rayon. For the treatment group, the pad contained four antioxidant medicinal plant extracts including *Schizonepeta tenuifolia var*. *japonica Kitagawa*, *Angelica dahurica Bentham ET hooker*, *Rehmannia glutinosa Liboschitz var*. *purpurea Makino*, *and Cassia tora L*. and nothing for the placebo group.^12^ Each treatment session was 15 min long and both groups underwent a three treatment sessions daily for 8 weeks. During the study, the participants used the same pad. Examinations were performed at baseline, 4, and 8 weeks after beginning the treatment. At each visit, each subject underwent a detailed ocular examinations including best corrected visual acuity, slit lamp biomicroscopy, OSDI score, tear film BUT, Schirmer’s test, urine human chorionic gonadotropin, vital sign, and history of additional usage of eye drops which can affect ocular surface. Participants were instructed to wear the glasses in the early morning, midafternoon and late evening. We tested participants at 10 to 11 AM at the each visit. Therefore, there are two or three hour differences between wearing of glasses and clinical tests. All patients were also questioned regarding any ocular symptoms related to the glasses at all visits for safety reasons.

### Outcome assessment

The primary outcome measure was the change in subjective symptoms of DED. The subjective symptoms were graded using the OSDI score (0 to 100), with higher scores representing greater disability.[13] The secondary outcome measures were tear film BUT and Schirmer’s test (with anesthesia). These tests were performed as described previously by a single investigator (KCY) who was blind to the patient’s clinical informations. [14, 15] In brief, 2 μl of 1% fluorescein solution was instilled on to the inferior palpebral conjunctiva. The interval between the last blink and the appearance of the first precorneal hypofluorescent spot, streak, or other irregularity interrupting the normal homogenous fluorescein pattern was recorded as the tear film BUT (seconds).[14] Schirmer’s test was performed by instilling one drop of proparacaine 0.5% anesthetic, waiting for 5 min. A standard Schirmer test strip was then placed in the lateral canthus for another 5 min with the eyes closed. The length of wetting of the strip was measured using the millimeter scale.[15] Compliance was determined on the basis of self-daily records of wearing time by minute based on 15 minutes per once, 3 times a day for 8 weeks. All participants included in the analysis except for a drop-out participant. Compliance was monitored by the examiner at the time of each visit through the wearing time diary.

### Statistical analysis

Sample size and power calculations were based on the primary efficacy endpoint. Results from previous study was used to estimate the mean change expected [16]. Sample size was determined by requiring 80% power and a 0.05 alpha level (one sided) in detecting a difference between the baseline and post treatment with OSDI score of approximately 17.3, based on Wilcoxon’s signed rank test. Twenty five patients were included in each group considering the dropout rate approximately 20%.

SPSS 17.0 software (SPSS Inc., Chicago, IL) was used for statistical analysis. Shapiro-Wilk test revealed non-normal distribution of data, hence Wilcoxon rank sum and Mann-Whitney U test were used to compare the results, with post hoc Bonferroni test to correct for multiple comparisons; the *P*-value for these two tests were 0.017 because there were three comparisons involved (0.05/3). Chi square test was used to compare gender distribution. Medians and interquartile ranges were described. Repeated measure analysis of variance (ANOVA) was instituted to test the differences between groups over time. To check the assumptions for repeated measures ANOVA, we used Mauchly’s Test of Sphericity and Box’s Test of Equality of Covariance. *P* < 0.05 was considered statistically significant.

## Results

### Patient demographics and tear film and ocular surface parameters

**Fig 2:**
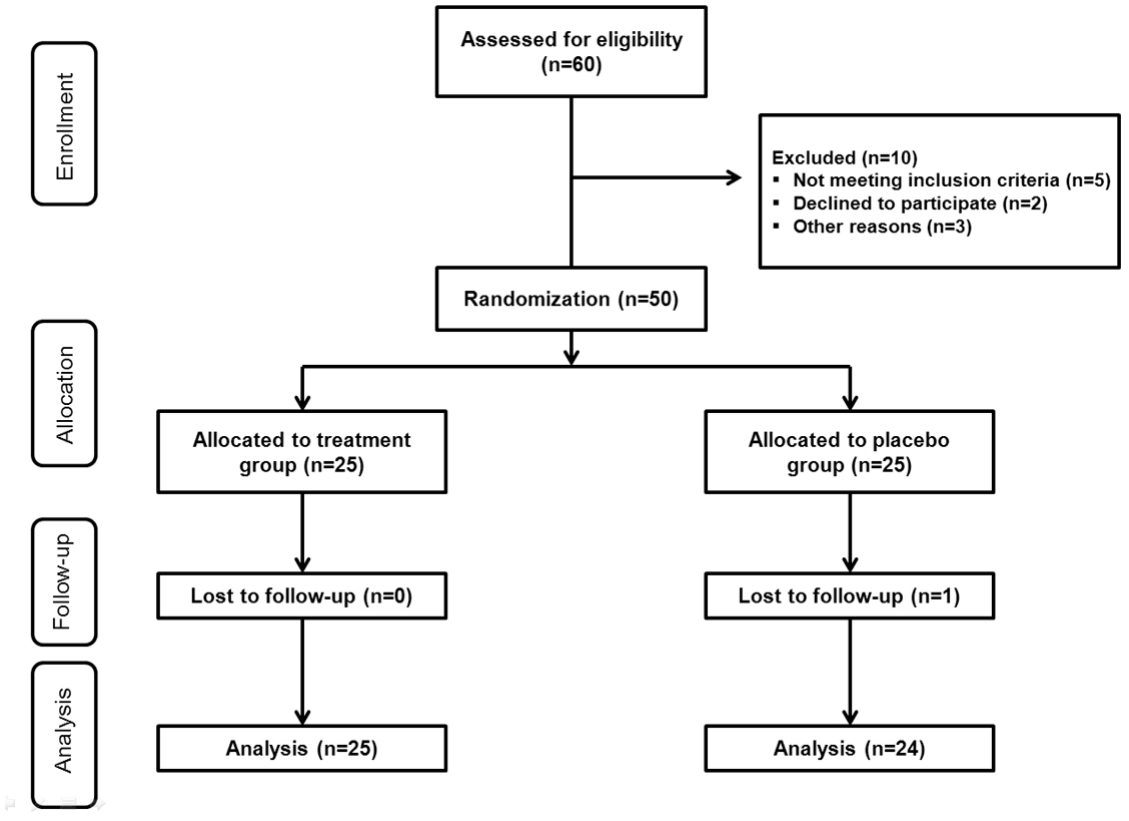
Flow chart showing enrollment, allocation, follow up, and analysis of participants. OSDI scores of the treatment and placebo groups are shown in Table 1.

Fifty patients with DED met the inclusion criteria and were randomly assigned to two groups prior to the study initiation, the treatment (n = 25) or placebo groups (n = 25). The randomization was performed according to a computer-generated randomization list (Fig 2). One participant from the placebo group was dropped from the study because of follow up loss. The number of participants was 24 (12 treatment, 12 placebo groups) in Chonnam National University hospital and 25 (13 treatment, 12 placebo groups) in Chung-Ang University Hospital, respectively. There were no differences in the use of glasses between two groups during the study. Among 25 patients in the treatment group, 11 were men and 14 were women, and the mean age was 24.84 ± 4.99 years. Among 24 patients in the placebo group, 15 were men and 9 were women, and mean age was 25.88 ± 5.75 years. Compliance rates in the treatment and placebo groups were 98.26 ± 2.64% and 97.56 ± 3.99%, respectively. There were no significant differences in age (*P* = 0.504), gender (*P* = 0.256), and compliance (*P* = 0.476) between the groups.

**Table 1:** Ocular surface disease index scores in treatment and placebo groups at baseline, 4 weeks, and 8 weeks after treatment [median (interquartile range), points].

	Follow-up periods			*P value* *		
	Baseline	4 weeks	8 weeks	*P*1	*P*2	*P*3
Treatment group (N = 25)	29.16 (27.08–31.25)	20.83 (18.18–27.08)	18.72 (16.67–22.90)	< .001	0.011	< .001
Placebo group (N = 24)	28.12 (19.26–29.54)	25.00 (20.11–27.08)	25.00 (18.75–32.56)	0.192	0.884	0.615
*P* value^†^	0.565	0.455	< .001			

*P*1 baseline vs. 4 weeks; *P*2 baseline vs. 8 weeks; *P*3 4 weeks vs. 8 weeks

* Wilcoxon signed rank test with Bonferroni correction (*P*<0.017)

^†^ Mann-Whitney test with Bonferroni correction (*P*<0.017)

Although there was no significant improvement in OSDI scores in the placebo group during the study, the treatment group showed significant improvement at 4 and 8 weeks of follow-up period (all *P* < 0.001). In addition, intergroup comparison revealed that OSDI score was significantly lower in treatment group at 8 weeks of follow-up (*P* < 0.001).

Table 2 shows the tear film BUT of both groups during follow-up period. Compare to baseline, the treatment group showed significant differences at 4 weeks (*P* < 0.001) and 8 weeks (*P* < 0.001) after treatment, whereas the placebo group showed no significant differences. However, the intergroup comparison showed no significant differences.

**Table 2:** Tear film break up time in treatment and placebo groups at baseline, 4 weeks, and 8 weeks after treatment [median (interquartile range), seconds].

	Follow-up periods			*P value* *		
	Baseline	4 weeks	8 weeks	*P*1	*P*2	*P*3
Treatment group (N = 25)	3.5 (2.0–5.4)	5.0 (3.5–7.0)	5.4 (4.0–6.6)	< .001	< .001	0.058
Placebo group (N = 24)	4.0 (3.0–6.1)	4.0 (3.0–5.9)	4.5 (4.0–6.2)	0.888	0.623	0.519
*P* value^†^	0.251	0.299	0.125			

*P*1 baseline vs. 4 weeks; *P*2 baseline vs. 8 weeks; *P*3 4 weeks vs. 8 weeks

* Wilcoxon signed rank test with Bonferroni correction (*P*<0.017)

^†^ Mann-Whitney test with Bonferroni correction (*P*<0.017)

As shown in Table 3, the results of the Schirmer’s test showed significant improvement at 4 weeks compare to baseline in the treatment group (*P* = 0.035), while no significant change was observed in placebo group. No significant differences were observed between two groups during follow-up.

**Table 3:** Schirmer’s test value in treatment and placebo groups at baseline, 4 weeks, and 8 weeks after treatment [median (interquartile range), millimeters].

	Follow-up periods			*P value* *		
	Baseline	4 weeks	8 weeks	*P*1	*P*2	*P*3
Treatment group (N = 25)	6.4 (5.0–8.5)	7.4 (6.5–9.2)	7.0 (5.1–8.9)	0.035	0.314	0.186
Placebo group (N = 24)	6.8 (5.2–8.4)	6.7 (5.0–8.4)	7.2 (6.3–8.7)	0.862	0.127	0.375
*P* value^†^	0.695	0.315	0.505			

*P*1 baseline vs. 4 weeks; *P*2 baseline vs. 8 weeks; *P*3 4 weeks vs. 8 weeks

* Wilcoxon signed rank test with Bonferroni correction (*P*<0.017)

^†^ Mann-Whitney test with Bonferroni correction (*P*<0.017)

The OSDI and tear film BUT showed a significant improvement in the treatment group between baseline and 4 and 8 weeks after treatment. However, in the comparison between the treatment and the placebo groups, there were no significant differences at the time of each visit except for 8 weeks after treatment in OSDI score.

In addition, repeated measure ANOVA demonstrated significant changes between the two groups over time in OSDI score (*P* = 0.002) and tear film BUT (*P* = 0.015) However, there was no significant change in Schirmer’s test (*P* = 0.320). During the study, no adverse effects were reported in both treatment and placebo groups.

## Discussion

Recently, it has been demonstrated that oxidative stress as well as inflammation in the lacrimal functional unit plays an important role in the pathogenesis of DED.[17–19] Increased levels of oxidative stress markers and production of ROS in the corneal epithelium of blink-suppressed dry eyes has been reported.[18] Levels of lipid oxidative stress markers increased in the tear film and conjunctiva of dry eye patients with Sjögren’s syndrome and were correlated with disease severity.[19] In addition, a close relationship has been found between ROS production, lipid peroxidation-related membrane damage, and inflammation in DED.[19] Several antioxidants such as green tea polyphenols and omega-3 fatty acids have shown anti-inflammatory and anti-oxidative effects in human corneal epithelial cells, and topical or systemic use of these agents can decrease clinical signs and inflammatory markers in the tear film, ocular surface, and lacrimal gland of dry eye.[6–10]

In our previous study, we already demonstrated the efficacy of a mixture of four natural plant EtOH extracts in protecting human corneal epithelial cells from oxidative stress induced by irradiation with short wavelength LED.[12] In our experiments, the increased mRNA and protein expression of the antioxidant enzymes heme oxygenase-1, peroxiredoxin-1, catalase, and superoxide dismutase-2 in corneal epithelial cells was induced by medicinal plant EtOH extracts and increased in a dose dependent manner. Interestingly, the extracts of medicinal plants could inhibit the amount of ROS produced after short wavelength LED irradiation.[12] We also demonstrated that the mixture of extracts had a higher IC50 value compared with the individual EtOH extracts.[12] These findings suggested additive effects of these individual extracts that could make the mixture more potent.

In the present study, we used identical EtOH extracts of medicinal plants to demonstrate the antioxidant effects in patients with mild DED. We first evaluated the clinical safety and efficacy of the wearable glasses containing mixtures of medicinal plants which act as an antioxidants using prospective, multicenter, double-blind, randomized, placebo-controlled trial. OSDI score and tear film BUT significantly improved in the treatment group at 4 and 8 weeks after wearing glasses. Compare to the placebo group, the OSDI score was significantly lower in the treatment group at 8 weeks. In addition, repeated measure ANOVA showed significant changes between the groups depending on the time in OSDI score and tear film BUT. These findings suggested that glasses containing mixed medicinal plant extracts used in this study can provide subjective and objective improvement of DED via the antioxidant defense system.

Our findings are comparable to the results of previous studies that have demonstrated the efficacy of topical or systemic antioxidant agents in improving signs and symptoms of DED.[9, 10] Topical omega-3 essential fatty acids and hyaluronic acid mixtures reduced corneal irregularity and epithelial barrier disruption, and decreased levels of inflammatory cytokines and oxidative stress markers on the ocular surface in the murine model of desiccating stress-induced dry eye.[9] In addition, oral consumption of omega-3 fatty acids has been associated with a decrease in the rate of tear evaporation and an improvement in OSDI score.[10] Furthermore, Kim et al[20] have reported that topical vitamin A eye drops in patients with DED contributed to improvements in blurred vision, tear film BUT, Schirmer test, and impression cytologic findings. The four medicinal plant EtOH extract used in the present study has volatile properties that allowed the antioxidant substances to be transmitted to the ocular surface while patients were wearing the glasses.

It has been shown that oxidative stress reduces aqueous tear production and that antioxidants increase Schirmer scores.[21] However, Blades et al[22] reported that tear stability was significantly improved following 1 month of oral antioxidant complex intake, although tear volume was not improved in patients with DED. In the present study, Schirmer’s test in the treatment group showed significant difference only between baseline and 4 weeks, and differences in the other comparisons were not. In addition, there were no significant changes depending on the time and between treatment and placebo groups. A possible explanation of this observation could be that the antioxidant substances were transmitted by diffusion only to the ocular surface and not to the lacrimal gland. Another possibility is that the medicinal effect of the extract in the pad getting weaker over time as a function of half-life.

Our study had several limitations. First, we did not conduct analyses of the amount of antioxidant substances that reached the ocular surface from the glasses pads and the changes in levels of oxidative stress markers in the ocular surface. Second, our study included only patients with mild DED according to OSDI score. Third, the clinical efficacy of the glasses was not compared with other dry eye treatment options such as cyclosporine, steroids, or artificial tears. Future studies are needed to evaluate the effects of antioxidant containing glasses for patients with moderate to severe DED and to compare the glasses with other treatment options. Additionally, further researches are needed to assess changes in levels of antioxidant materials over time for evaluating validity duration and analyze chemical antioxidant composition of the four natural plant EtOH extracts.

In conclusion, wearing antioxidant glasses containing extracts of medicinal plants improve dry eye symptoms as well as clinical parameters of DED as indicated by a positive trend in primary and secondary outcome measures. This therapy may be used as adjunctive therapeutic option for the treatment of DED.

## Supporting Information

S1 CONSORT Checklist CONSORT Checklist. (DOC)

S1 Dataset Dataset. (XLSX)

S1 Protocol Protocol (English). (DOCX)

S2 Protocol Protocol (Korean). (DOCX)
