# Supplementary material for: Clinical Effect of Antioxidant Glasses Containing Extracts of Medicinal Plants in Patients with Dry Eye Disease: A Multi-Center, Prospective, Randomized, Double-Blind, Placebo-Controlled Trial
Source: PLoS One. 2015 Oct 12;10(10):e0139761. doi: 10.1371/journal.pone.0139761 (PMC4601690; doi:10.1371/journal.pone.0139761)
Supplement: S1 Protocol — (DOCX) [file pone.0139761.s003.docx]

**Clinical study protocol**

**Clinical Effect of Antioxidant Glasses Containing Extracts of Medicinal Plants in Patients with Dry Eye Disease**

Protocol version: 2.3

Protocol Date: 28 September 2012

Project Leader: Kyung Chul Yoon, M.D., Jae Chan Kim, M.D.

1. Protocol title

Clinical effect of antioxidant glasses containing extracts of medicinal plants in patients with dry eye disease

2. Objective

To investigate the clinical efficacy and safety of wearable antioxidant glasses containing extracts of medicinal plants in patients with mild dry eye disease.

3. Background

Currently, the traditional therapies for DED are life style modification such as home humidification and avoidance of infrequent blinking, topical lubrication with artificial tears, anti-inflammatory and immune-modulatory eyedrops, or surgical treatment including temporal or permanent punctal occlusions. Lubrication with artificial tears on a daily basis is the most recommended way to treat dry eye, however, this method has several limitations. Natural tears have a complex composition of water, salts, hydrocarbons, proteins, and lipids, which artificial tears cannot completely substitute. In addition, the integrity of the three-layered lipid, aqueous, and mucin structure, vital to the effective functioning of the tear film, cannot be reproduced by these artificial components. Furthermore, artificial tears are delivered intermittently, rather than continuously as are natural tears. Moreover, in the cases of preservatives containing artificial eyedrops, even though the concentration of preservatives in artificial tear preparations is generally low, their prolonged presence on an already compromised ocular surface, such as that of a dry eye, can cause serious iatrogenic effects, worsening the ocular surface diseases.

Surgical treatment of DED could be performed by temporary or permanent closure of lacrimal punctum. Occlusion of the lacrimal puncta or canaliculi prevents the drainage of natural and artificial tears and is currently the most common surgical therapy for DED. However, applying to the patients with mild DED are difficult to access due to high cost, irreversibility, side effects, and pain related to the surgical procedure.

Therefore, in this prospective, multicenter, double-blind, randomized, placebo-controlled clinical trial, we evaluated the clinical effects and safety of the wearable glasses containing mixtures of anti-oxidant medicinal plants in patients with mild DED who concerned about side effects related to long term use of artificial tears and difficult to apply the surgical treatment.

4. Study design

A Multi-Center, Prospective, Randomized, Double-Blind, Placebo-Controlled Trial: Mixed medicinal plants extracts containing glasses vs placebo glasses

5. Method

Patients were randomly assigned to either treatment or placebo groups. Patients of treatment group received a commercially produced glasses and those of placebo group received placebo glasses. Both treatment and placebo glasses were supplied in identical containers for masking purposes. The treatment pad contains four anti-oxidant medicinal plants extracts including *Schizonepeta tenuifolia var. japonica Kitagawa, Angelica dahurica Bentham ET hooker, Rehmannia glutinosa Liboschitz var. purpurea Makino, and Cassia tora L*. Each treatment session was 15 min long and both groups underwent a treatment session three times a day for 8 weeks. Subjects were seen at baseline, 4 weeks, and 8 weeks after beginning the treatment. At each visit, each subject underwent a detailed ocular examinations including best corrected visual acuity, slit lamp biomicroscopy, OSDI score, tear film BUT, Schirmer’s test, urine HCG, vital sign, and history of additional usage of eyedrops which can affect ocular surface. All patients were also questioned regarding any ocular symptoms related to the glasses at all visits for safety reasons.

6. Inclusion criteria

(1) Patients aged 20 to 60 years

(2) One or more dry eye-related ocular symptoms (> 3 months) such as dryness, irritation, and burning sensations

(3) Ocular Surface Disease Index (OSDI) score of 13 to 32 (mild to moderate)

(4) Tear film break-up time (BUT) of <10 s or Schirmer’s test (with application of local anesthetic) value <10 mm for 5 min.

7. Exclusion criteria

(1) patients with pregnant woman

(2) active eye and periocular skin inflammation

(3) vitamin A deficiency

(4) previous ocular surgery within 3 months before the study

(5) history of wearing contact lenses

(6) history of active treatment for dry eyes such as punctal occlusion or the usage of anti-inflammatory eye drops (topical steroid or topical cyclosporin) within 1 month of the study

(7) systemic condition or medication that could cause dry eye.

8. Statistics

Power calculation is based on “Acupuncture Reduces Symptoms of Dry Eye Syndrome: A Preliminary Observational Study”. It takes 25 patients in each group to show 17.3 point difference between the groups in Ocular surface disease index (OSDI) score, with 80% power and significance level 0.05.

9. Outcome measurements

(1) OSDI score

The OSDI questionnaire were graded on a scale of 0 to 4, where 0 indicates none of the time; 1, some of the time; 2, half of the time; 3, most of the time; and 4, all of the time. The total OSDI score was then calculated on the basis of the following formula: OSDI = [(sum of scores for all questions answered) × 100] / [(total number of questions answered) × 4]. Thus, the OSDI is scored on a scale of 0 to 100, with higher scores representing greater disability.

(2) Tear film break-up time

Tear film BUT was evaluated 2 µl of 1% fluorescein solution was instilled on to the inferior palpebral conjunctiva after gentle depression of the lower eyelid. The interval between the last blink and the appearance of the first precorneal hypofluorescent spot, streak, or other irregularity interrupting the normal homogenous fluorescein pattern was recorded as the tear film BUT (seconds).

(3) Schirmer test

Schirmer’s test was performed by instilling one drop of proparacaine 0.5% anaesthetic, waiting for 5 min. A standard Schirmer test strip was then placed in the lateral canthus for another 5 min with the eyes closed. The length of wetting of the strip was measured using the millimeter scale.

10. Referances

1. Lemp MA. New strategies in the treatment of dry- eye states. Cornea 1999;18:625–32.

2. Murube J, Paterson A, Murube E. Classification of artificial tears. I: Composition and properties. Adv Exp Med Biol 1998;438:693–704.

3. Pflugfelder SC. Advances in the diagnosis and management of keratoconjunctivitis sicca. Curr Opin Ophthalmol 1998;9:50–3.

4. Lopez Bernal D, Ubels JL. Quantitative evaluation of the corneal epithelial barrier: effect of artificial tears and preservatives. Curr Eye Res 1991;10:645–56.

5. Murube J, Murube E. Treatment of dry eye by blocking the lacrimal canaliculi. Surv Ophthalmol 1996;40:463–80.
