# Supplementary material for: Clinical Effect of Antioxidant Glasses Containing Extracts of Medicinal Plants in Patients with Dry Eye Disease: A Multi-Center, Prospective, Randomized, Double-Blind, Placebo-Controlled Trial
Source: PLoS One. 2015 Oct 12;10(10):e0139761. doi: 10.1371/journal.pone.0139761 (PMC4601690; doi:10.1371/journal.pone.0139761)
Supplement: S2 Protocol — (DOCX) [file pone.0139761.s004.docx]

**연구계획서**

**1. 임상시험의 명칭 및 단계**

**1.1. 명칭 : 안구건조증 임상시험용 의료기기의 안전성 및 유효성 평가**

| **번호** | **기 관 명** | **소 재 지** | **전 화** | **팩 스** |
| --- | --- | --- | --- | --- |
| 1 | 전남대학교병원 | 광주광역시 동구 제봉로 671 | 062-220-5114 | 062-224-7821 |
| 2 | 중앙대학교병원 | 서울 용산구 한강로3가 65-207 | 02-748-9838 |  |

**2. 임상시험실시기관의 명칭 및 소재지**

**3. 임상시험의 책임자․담당자 성명 및 직명**

**3.1 시험책임자**

| **번호** | **성 명** | **소속 기관명** | **전 공** | **직 위** | **전 화** |
| --- | --- | --- | --- | --- | --- |
| 1 | 윤경철 | 전남대학교병원 | 안과 | 부교수 | 062-220-6684 |
| 2 | 김재찬 | 중앙대학교병원 | 안과 | 교수 | 02-748-9838 |

**4. 임상시험용 의료기기관리자 성명 및 직명**

| **번호** | **성 명** | **소속 기관명** | **전 공** | **직 위** | **전 화** |
| --- | --- | --- | --- | --- | --- |
| 1 | 윤경철 | 전남대학교병원 | 안과 | 부교수 | 062-220-6684 |
| 2 | 김재찬 | 중앙대학교병원 | 안과 | 교수 | 02-748-9838 |

**5. 임상시험의뢰자의 성명 및 주소**

**5.1 의뢰자**

| **회사명** | **대표이사** | **소 재 지** | **전 화** |
| --- | --- | --- | --- |
| 비엠생명공학연구소(주) | 김 희 구 | 순천시 해룡면 호두리 율촌제 1지방산  단 6블럭 전남테크노파크 벤처동 321호 | 062-974-0771 |

**6. 임상시험실시 기관 및 역할**

**6.1. 협연기관(coordinating center)**

| **번호** | **기 관 명** | **총괄책임연구자** | **전 공** | **직 위** | **전 화** |
| --- | --- | --- | --- | --- | --- |
| 1 | 전남대학교병원안과 | 윤 경 철 | 안 과 | 부교수 | 062-220-6684 |

**6.2. 협연기관의 역할**

1) 임상시험 추진관리

임상시험설계 단계에서부터 연구형태를 설계하고, 적절한 연구대상자 수를 산출하며 임상시험 추진일정을 수립, 공동연구기관과 협의 임상시험을 추진한다. 또한 피험자가 임상시험용 의료기기를 임상계획서에 따라 적정하게 착용할 수 있도록 관리한다.

2) 검사방법의 표준화

공동연구기관과 협의하여 연구계획서에 따라 피험자선정기준, 검사기준과 방법 등을 연구기관 간에 표준화될 될 수 있도록 조정 관리한다.

3) 무작위배정방법수행

임상시험용 의료기기를 무작위배정 방법에 따라 피험자에게 배정 관리하고 임상시험완료 후 시험용 의료용구를 분해, 패드 유무에 따라 시험군과 대조군으로 분류하는 역할을 수행한다.

4) 통계분석 수행 및 데이터베이스 관리

임상기간 동안 공동연구기관의 검사 자료의 수집과 편집, 분석에 대한 계획을 수립하고 수집된 검사 증례기록서 토대로 데이터베이스를 구축, 임상계획서에 기술된 통계방법에 따라 분석한다. 또한 연구 자료를 검토하여 문제가 발견되면 해당 기관으로 보내어 수정보완하게 하고, 통계적으로 분석하는 기능을 수행함으로써 연구의 객관성과 타당성을 보장할 수 있게 한다.

5) 임상시험 후 추적관찰

임상계획서에 의거 탈락된 환자에 대한 리스트를 작성하고, 처치에 대한 업무를 수행하며 임상시험 종결 후, 추적관찰이 가능한 환자들에 대한 추적관찰 기간 동안 소재지 파악 수집되는 자료 파일을 유지관리 기능을 담당한다.

**7. 임상시험의 배경 및 목적, 추진절차**

**7.1. 임상시험 배경**

**1) 배경**

최근에는 안구건조증의 고전적인 치료법으로 생활환경 가습, 눈 깜빡임 횟수의 증가 등의 생활습관 변화와 인공눈물, 항염증 및 면역억제 안약과 일시적 또는 영구적인 눈물점 폐쇄 등의 수술적 방법이 이용되고 있다. 안구건조증을 치료하는 방법으로 매일 인공눈물을 점안하는 방법이 가장 많이 이용되고 있으나, 몇 가지 단점이 있다. 자연적으로 생성되는 눈물에는 물, 염분, 탄화수소, 단백질과 지방 등 인공눈물에서는 함유하지 못하는 차이점이 있다. 게다가 지방층, 수성층, 뮤신층의 3개 층이 눈물막에 필수적인 기능을 하는데, 인공눈물은 이것을 대체할 수 없다. 또한, 자연적으로 나오는 눈물은 계속해서 나오는데 반해 인공눈물은 간헐적으로 점안한다. 더군다나 보존제를 함유하는 인공눈물은 그 양이 극소량이라고 하더라도 지속적으로 안구표면에 노출이 되게 되면 안구건조증이나 독성 결막염 같은 심각한 합병증을 일으키게 된다.

수술적 치료는 일시적 또는 영구적으로 눈물점 폐쇄를 시행할 수 있다. 눈물점이 폐쇄되면 자연적으로 생성되거나 인공 눈물이 빠지는 것을 막을 수 있어서 안구건조증에서 가장 많이 시행되는 수술적 치료이다. 하지만 경증의 안구건조증에서는 비싼 비용, 되돌릴 수 없는점, 부작용, 통증 등으로 인해 널리 시행되지 못하는 실정이다.

따라서 본 연구에서는 인공 눈물을 장기간 사용하였을때 부작용이 우려되고 수술적 치료를 시행하기에는 경한 안구건조증 환자에서 항산화물질을 함유한 약초가 들어있는 안경을 착용하는게 효과적이면서 안전한지 알아보고자 한다.

2**) 임상시험용 의료기기의 선행연구**

BM생명공학연구소(주)에서 자석패드가 부착된 안구건조증치료용 의료용자기발생기를 개 발하여, 본 임상시험의 협연센터인 전남대학교병원 안과에서 2009년 10월 1일 ~ 2010년 3월 31일까지 본 임상시험용 의료기기인 EPA - Alpha(α)를 가지고 착용전후 비교평가를 선행연구 하였다. 건성안(안구건조증)이 없는 27명의 정상안의 대상자 중 남자는 8명, 여자는 19명이었고, 평균나이는 28.3±8.1세(22-55세)였다. 이들 정상인에서 EPA - Alpha(α) 시작, 2주, 4주 및 8주째 대상자의 증상, 눈물막파괴시간, 기초눈물분비량, 눈물청소율검사, 각막감각, 각막상피병증의 정도, 결막 술잔세포의 수, 편평상피화생의 정도 등의 눈물막 및 안구표면 인자, 시력과 안압의 변화를 조사한 결과 시력과 안압 등에서는 안전성이 있는 것으로 평가되었으며, 눈물의 양이 변화는 것으로 나타나 안구건조증 환자를 대상으로 한 임상시험 검정이 필요하게 되었다.

**7.2 임상시험의 목적**

안구건조증환자에게 안구건조증 임상시험용 의료용구를 8주 동안 착용케 하여 1차변수인 안구표면질환지수, 눈물막파괴시간, 기초눈물분비량을 통하여 착용하기 전과 착용 후의 수치변화를 비교 평가하여 안구건조증 치료용 의료기기로서의 안전성과 유효성을 평가 하는데 그 목적이 있다.

**7.3 임상시험 추진절차**

피험자모집 공고 - 피험자 선정기준 적합 여부판정(1차검사 : OSDI 자가평가) - 임상시험 주의사항 설명 및 계약 - 시험용 의료기기배정 - 임상시험검사(시작, 4주차, 8주차) -

시험군 대조군분류 - 통계평가 및 결과보고 순으로 한다.

**8. 임상시험용 의료기기의 원자재 및 제형(형상/구조/수치)등**

8**.1 의료기기의 특징**

본 EPA - Alpha(α)의 임상시험용 의료기기는 자석패드가 들어 있는 의료용자기발생기로, 안면부에 탈부착이 용이한 고굴 형태의 외부지지대로 구성되어 있다. 외부지지대는 안구 주위를 감쌀 수 있도록 설계되어 있으며, 자석패드는 이 외부지지대 안에 부착되어 있어, 자석은 안구와 직접적으로 접촉할 수 없는 안구비접촉식 제품이다. 또한 본 의료용자기 발생기는 자기장의 자파를 이용하여 안구주변의 혈점을 자극, 혈액순환을 도와 눈물의양 과 질을 호전시켜 안구건조증 치료에 영향을 주는 것으로 가정에서 사용하는 의료용자기 발생기 2등급이다.

**8.2 원자재 및 제형(형상/구조/수치)**

1) 품 목 명 : 의료용자기발생기(A85010.01. 2등급)

2) 형명(모델명) : EPA – Alpha(α)II

3) 성 능 : 안구건조증 환자의 안구건조증병변 증상 호전

① 안구표면질환지수(OSDI) 개선

② 눈물막파괴시간(BUT) 연장

③ 눈물분비량 호전

5) 색 상 : 바이올렛

6) 진짜 시험용 의료기기와 가짜시험용 의료기기의 차이점

진짜 시험용 의료기기와 가짜시험용 의료기기는 모두 동일 색상, 동일 형태의 제품으 로 육안식별이 불가능하여 진짜 가짜의료기기를 구별할 수 없는데, 대조군이 사용하는 가짜 시험용 의료기기는 자석패드 대신에 자석패드와 똑 같은 형태와 같은 자석이 없 는 가짜 부직포가 들어 있다.

7) 사용방법

임상시험용 의료기기는 임상시험기간 동안 사용하며, 눈을 감은 상태로 일회 15분/일일 3회(오전,오후,저녁)착용하며, 착용 후 1분간 안구를 상하좌우로 눈 운동을 한다.

**9. 대상 질환**

**9.1. 안구건조증 증상**

1) 책이나 가까운 물체를 잠시만 바라봐도 눈이 침침하고 머리가 무거워진다.

2) 감정적으로 스트레스를 받았을 때 울기가 힘들다.

3) 콘택트렌즈 착용이 어렵다.

4) 가끔 옆구리가 결리거나 아픈 증상이 있다.

5) 건조한 곳이나 오염이 심한 곳에서 가끔 눈이 화끈거린다.

6) 계속적으로 눈이 까칠 까칠하거나 통증이 있다.

7) 실 같은 분비물이 나온다.

8) 눈이 몹시 깔깔하고 눈알이 아프다.

9) 오후만 되면 눈이 팽팽해지고 눈앞이 흐릿하다.

10) 다른 사람 눈을 정면으로 쳐다보기 어렵다.

11) 지속적인 시각 집중이 필요한 독서, 컴퓨터 작업이 견디기 어려워진다.

12) 빛에 비정상적으로 예민해지고 극심한 통증을 호소한다.

**9.2. 특징**

안구건조증 환자로 눈물의 량이 부족하고 문물막파괴시간이 짧아 눈알이 뻑뻑하며 심한경우 눈이 충혈되어 일상생활이 심히 불편함.

**10. 피험자의 선정기준, 제외기준, 목표한 피험자의 수 및 근거**

**10.1. 선정기준**

1) 20-60세 사이의 성인 남녀

2) 임상시험에 참여하고자 하는 피험자 중에서 안구표면질환지수(Ocular surface disease Index, OSDI)가 13-32점 이상에 해당하는 자

3) 임상시험에 참여하고자 하는 피험자 중에서 안구건조증 환자군에 속하는 눈물분비량 (Schirmer score, BST) 측정이 10mm/5분 이하에 해당하는 자

4) 임상시험에 참여하고자 하는 피험자 중에서 안구건조증 환자군에 속하는 눈물막파괴 시간(Tear film break-up time, BUT)이 10초 이하에 해당하는 자

**10.2. 제외기준**

(1) 임산부

(2) 눈이나 눈주변 피부에 활동성 염증병변

(3) 비타민 A 결핍

(4) 3개월 이내에 눈 수술을 받은자

(5) 콘택트 렌즈 착용자

(6) 1개월 이내에 눈물점 폐쇄나 스테로이드 싸이클로 스포린 점안액 등 안구건조증 치료를 받은자

(7) 안구건조증을 유발할만한 전신적 상태 또는 약물 복용자

**10.3. 피험자의 수 및 산출근거**

1**) 피험자 수**

(1) 피험자 모집인원 : 총 50명

| 대상 | 전남대학교병원 | 중앙대학교병원 | 합계인원 |
| --- | --- | --- | --- |
| 인원 | 25 | 25 | 50 |

**2) 산출근거**

본 임상시험에서 필요한 피험자 수 산출은 “Acupuncture Reduces Symptoms of Dry Eye Syndrome: A Preliminary Observational Study”에 근거하였다. 유의수준 5%, 검정력 80%를 확보하기 위해서는 OSDI 점수가 17.3점 차이가 있다고 가정하였을 때 공식에 의하여 25명이라고 결정할 수 있다

- 귀무가설 : 시험군과 대조군의 안구건조증병변 호전정도는 동일하다(두 군 모두 임상시 험용 의료기기를 착용하기 전 안구건조증병변 검사 수치일 것이다).

- 대립가설 : 시험군과 대조군의 안구건조증병변 호전정도는 다르다(대조군의 임상시험용 의료기기를 착용하기 전 안구건조증병변 검사 수치보다 우수한 00일 것이 다)

배정비가 1:1인 연구에서 제1종 오류 α와 제2종 오류 β를 고려하여 각 군당 필요한 연구 대상수는 다음을 이용하여 산출할 수 있다.


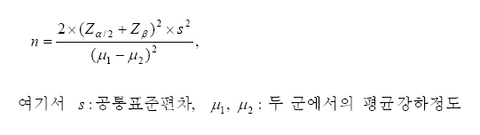


**11. 무작위배정, 눈가림법, 비교군설정 등**

**11.1. 시험용 의료기기 무작위배정(randomization)**

임상시험연구자가 치료방법 또는 검정에 대한 의지가 개입되지 않도록 하기위하여 피험자가 사용하는 시험용 의료기기 배정은 1 : 1 고정성 무작위배정(fixed randomiza -tion) 방법에 따라 모든 피험자가 받게 되는 치료방법과 검정의 배정 확률이 임상시험 기간 동안 동일하게 되도록 한다.

임상시험의뢰기관인 BM생명공학연구소(주)에서 자석패드가 들어 있는 진짜 시험용 의 료기기와 자석패드가 없는 가짜 시험용 의료기기를 선택하는 확률이 같도록 동일색상, 동일형태로 제작, 혼합하여 박스에 넣어 밀봉 혼합하여 협연센터(전남대학교병원 안과) 에 제공, 임상시험실시기관의 임상시험책임자가 위의 시험용 의료기기를 수령, 피험자가 직접 시혐용 의료기기를 고를 수 있도록 한다.

**11.2. 양측 눈가림 (full double blind)에 의한 치료방법 시행**

피험자가 배정된 시험용 의료기기를 착용하여 임상시험 검사가 완료되기까지, 피험자 뿐 아니라 임상시험 관계자와 직접 접촉하는 모든 사람들, 예를 들면 연구수행과 자료 분석을 맡고 있는 역학 및 통계학자, 임상시험을 모니터하는 요원, 병원의 다른 의료진 등에게 임상시험 기간 동안 시험군 대조군을 알 수 없도록 한다. 통계를 진행하는 과정에서 시험군 대조군을 판별할 수 있도록 한다.

**11.3. 시험군 대조군 설정방법 및 설정시기**

피험자가 무작위배정방법에 따라 배정된 시험용 의료기기는 모두 동일 색상, 동일 형태 의 제품으로 진짜 가짜 육안식별이 불가능하여 임상시험기간 동안 시험군과 대조군으로 분류할 수 없다. 시험검사 완료 후 통계를 산출하기 위하여 공동연구기관(협연기관)에서 피험자 식별 번호와 착용 의료기기를 회수, 분해하여 자석패드 유무에 따라 시험군과 대조군으로 분류한다.

**12. 임상시험 기간 및 추진일정: IRB 승인일로부터 4개월**

1) 피험자 모집기간 : 2개월

2) 임상시험기간 : 2개월

3) 유효성 안전성 관찰기간 : 착용 후 2주, 4주, 8주

4) 임상적 평가 결과보고서 : 임상시험이 완료된 후 1개월 이내

**13. 임상시험방법(방법, 용법 등)**

**13.1 임상시험 방법**

**1) 검사 시기**

피험자로 선정되면 시험용 의료기기를 착용하기 전, 착용 후 4주, 8주에 한다.

**2) 검사 항목**

(1) 1차변수설정 : 안구표면질환지수(OSDI),눈물막파괴시간(BUT),기초눈물분비량

**3) 검사 방법**

(1**) 안구표면질환지수(OSDI) 검사**

설문지를 이용하여 아래와 같이 대상자의 증상(5항목), 기능(4항목), 유발인자(3항목)의 12항목을 0부터 4까지의 점수로 분류하여(증상이 없을 때를 0, 항상 있을 경우는 4), 0 부터 100까지의 점수로 표시한다.

① 지난 일주일 동안 다음과 같은 증상을 느낀 적이 있습니까?

가. 눈이 빛에 예민하다.

나. 눈에 모래가 들어간 것 같은 느낌

다. 눈이 따갑거나 쑤신다.

라. 시야가 흐리다.

마. 앞을 보기가 힘들다.

② 지난 일주일 동안 다음의 일상생활을 하는데 어렵고 힘든 적이 있습니까?

가. 독서

나. 야간운전

다. 컴퓨터 또는 현금지급기 사용

라. 텔레비전 시청

③ 지난 일주일 동안 당신의 눈이 다음의 상황에 처했을 때 불편한 적이 있습니까?

가. 바람이 부는 날씨

나. 건조한 장소

다. 에어컨이 가동되는 곳

(2**) 눈물막파괴시간(BUT) 검사**

눈물막 파괴시간을 측정은 적셔진 형광검사지를 결막구석에 접촉시킨 후 피검자가 수 초간 몇 번 동안 눈을 깜박이게 한다. 마지막으로 완전히 깜박인 후 눈을 감지 못하 도록 하고 그 시점부터 염색된 눈물막에서 검은 점 또는 구멍이 처음으로 발생할 때 까지의 시간을 세극등 현미경의 코발트블루 광원을 이용하여 3회 측정하여 그 평균값 을 초로 기록한다.

**(3) 기초눈물분비량(쉬르머 검사) 검사**

0.5% proparacaine hydrochloride (Alcaine, Alcon, USA)를 하측 결막낭에 넣고 5분 후 쉬르머 검사지(Eagle Vision, Memphis TN, USA)를 아래 눈꺼풀의 외측 1/3부분 에 5분간 접촉한 다음 젖은 부위의 길이를 재어 기본눈물분비량을 밀리미터 단위로 측정한다.

**13.2 임상시험용 의료기기 사용법**

1) 1일 3회 오전 오후 저녁에 사용하며, 1회 15분씩 착용한다.

2) 안면부에 시험용의료기기를 15분간 착용 후에는 안면부에서 탈착 후, 1분정도 안구를 좌우상하로 운동한다.

**14. 관찰항목/임상검사항목 및 관찰방법(통계분석방법)**

**14.1. 관찰항목**

1) 인구학적 조사 : 성별, 성명, 나이, 주민등록번호, 나이, 직업 등

2) 환자의 병력조사 : 안구건조증 증세 및 지속여부, 약물복용, 혈압 등.

3) 유효성. 안전성 관찰 :

(1) 유효성관찰

피험자의 임상시험용 의료기기 착용 전 검사, 착용 후 2 주째, 4주째, 8주째 안구표면질 환지수(OSDI), 눈물막파괴시간(BUT), 기초눈물분비량(쉬르머검사)을 하여 유의적 변화를 관찰한다.

(2) 안전성 관찰

시험용의료기기를 정상적인 사용방법에 따라 착용할 경우 발생하는 모든 의도되지 않은 유해사례(adverse event․adverse experience, AE),실마리정보(signal) 등을 포함하여 추적 관찰한다.

4) 임상적 평가

(1) 착용 전, 후 치료효과를 치료항목으로 비교

(2) 착용 후 합병증의 빈도

5) 대조군용 의료기기 및 시험군용 의료기기 성능비교

6) 기타 : 약물 복용 유무, 흡연력, 음주력 등

**14.2. 관찰검사 및 방법**

1) 피험자 동의서 서명

본 임상시험을 실시하기에 앞서, 피험자는 ‘피험자를 위한 설명서’에 관한 내용을 피험 자 본인 및 대리인에게 설명하고, 피험자 및 대리인이 내용을 잘 이해한 것을 확인한 다음, 자유의사에 따른 임상시험참가의 동의를 문서로 받는다. 또한 동의를 서명한 동 의서를 증례기록서에 기록한다. 단 20세 이하의 피험자는 법정대리인의 동의를 얻어 본인의 서명을 받는다.

2) 피험자 적합성평가

피험자선정 기준에 따라 검사 평가하며, 제외기준에 해당하는지 조사한다.

3) 피험자 식별코드부여

임상시험 참여에 동의하고, 피험자선정기준에 적합한 피험자에 한하여 피험자의 코드를 번호를 부여한다.

4) 피험자 식별코드는 다음의 방법에 따라 기입한다.

(1) 실시기관코드 : 전남대학교(CNUH)병원, 중앙대학교(CAUMC)병원

(2) 피험자식별코드 : 진료과 / 등록 된 순서 (D : 안과 / 01~80 : 번호)

(3) 모든 날짜는 다음과 같은 형식으로 기록한다.

ex : 2010년 06월10일 = 2010/06/10

**14.3. 통계분석 방법**

1) 배정된 대로 분석 원칙(intention-to-treat principle)에 따라 최초의 무작위배정상태를 그대로 유지 모든 피험자(탈락된 피험자 포함)를 분석군(시험군, 대조군)에 포함시켜 통계 분석한다.

2) 시험용 의료기기는 시험군과 대조군에서 검사결과 변화 비교에 대하여 독립 t-test로 T효과를 통계 분석한다.

3) 피험자가 시험용 의료기기를 착용하기 전, 4주, 8주 시점에 안구표면질 환지수(OSDI), 눈물막파괴시간(BUT), 기초눈물분비량(쉬르머검사)을 측정하여 반복측정 분산분 석(repeated measures analysis of variance, RM-ANOVA) 방법에 따라 시험군과 대조 군에서 안구건조증 증상 정도가 동일한지, 시간의 흐름에 따른 안구건조증 증상 정도 가 동일한지, 각 군에서 관찰된 안구건조증 증상 정도가 분포양상이 시간에 따라 다 른 패턴을 가지는지를 평가 비교분석 한다.

**15. 예측 부작용 및 사용상의 주의사항**

**15.1. 예측되는 부작용**

1) 본 임상시험용 의료기기 착용 후, 눈꺼풀 또는 각결막을 포함한 안구표면의 알레르기, 독성반응, 시력저하, 안압변화 등 합병증이 발생할 수 있다.

2) 시험용 의료기기 사용과 병용금기치료

임상시험기간 동안 피험자는 약물복용을 금기하며 또한 본 임상시험용 의료기기이외에 다른 안구건조증 치료관련 행위를 할 수 없다. 시험용 의료기기를 사용하는 임상기간 동안 안구건조증 질환이 악화되거나 다른 질환으로 피험자가 치료를 호소한 경우 예외 적으로 IRB 위원회에 보고 후 탈락으로 처리하며, 일반 환자치료에 준 한다. 이 경우에 는 임상시험 완료 후 시험용 의료기기를 분해, 가짜 진짜패드에 따라 통계 평가에 반 영한다.

**15.2 임상시험용 의료기기 사용시 주의사항**

피험자는 임상시험기간 동안 임상시험용 의료기기를 사용하기 전에는 다음의 사항을 주의하여야 한다.

1) 임상시험용 의료기기 사용시 손상이 발견된 경우에는 사용을 즉시 중지한다.

2) 신체에 이상을 느낀 경우에는 사용을 즉시 중지한다.

3) 임상시험용 의료기기 착용으로 닿는 안면부에 발진, 발적, 가려움 등의 증상이 나타난 경우에는 사용을 즉시 중지한다.

4) 임상시험용 의료기기는 피험자가 개조하지 않는다.

5) 임상시험용 의료기기의 파손으로 인한 부속품은 지정된 것을 사용하여야 한다.

**16. 중지, 탈락의 기준**

**16.1. 중지기준**

1) 임상시험의뢰자는 임상시험용 의료기기의 안전성 등의 사유로 임상시험을 중지하고 자 할 경우, 임상시험심사위원회에 임상시험 중지 요청을 하여 임상시험위원회의 결 정에 따라 임상시험을 중지 할 수 있다.

2) 발생된 이상반응 처치를 위하여 일시적으로 중지되는 경우.

3) 심각한 이상반응/이상 의료기기반응의 발생으로 중지되는 경우.

**16.2. 중지의 처리**

1) 임상시험이 중지 된 경우 임상시험책임자는 중지 된 시점까지 진행된 피험자에 대한 증례기록서, 임상시험 진행현황 및 결과를 정리하여 임상시험 의뢰자에게 전달하며 모든 시험 관련자료(증례기록서 및 임상시험의료기기)를 임상시험의뢰자에게 반납하 여야 한다.

2) 임상시험이 중지된 경우 임상시험책임자는 시험중지 사실과 함께 IRB위원회에 서면 으로 통보하며, 중지사유가 해제되었을 경우 IRB위원회의 승인을 받아 임상시험을 계속할 수 있다.

**16.3.** 탈락기준

1) 피험자 또는 법적 대리권자가 임상시험 참여 중단을 요청한 경우

2) 안전성, 유효성 평가에 영향을 줄 수 있는 수술, 약물 또는 다른 의료기기를 병행하 여 사용한 경우

3) 심각한 부작용이 발생한 경우

4) 치료방법을 제대로 수행하지 않는 경우

5) 피험자가 시험자의 지시에 불응하거나 또는 동의서에 제시된 사항을 준수 하지 않아 그 유효율의 평가에 영향을 미치는 경우

6) 임상시험과 관련이 없는 사유로 피험자가 사망한 경우

7) 기타 임상시험책임자가 임상시험 진행에 문제가 있다고 판단되는 경우

**16.4.** 탈락의 처리

1) 피험자가 중도 탈락 된 경우 탈락사유 및 탈락 전까지 진행된 임상시험 관련 자료와 배정된 시험용의료기기를 공동연구기관(협연센터:전남대학교병원 안과)에 제출한다.

2) 시험도중 피험자가 내원하지 못한 경우 피험자간의 건재 여부를 확인하고 그 이유를 분명하게 하여야 한다.

3) 중도 탈락된 자는 타당한 이유 또는 근거가 없는 한 안전성. 유효성의 통계분석에 반영된다.

4) 공동연구기관(협연센터:전남대학교병원 안과)에서는 탈락된 피험자의 시험용의료기기 를 분해하여 자석패드의 유무에 따라 시험군과 대조군으로 분류, 통계분석에 반영한 다.

**17. 유효성(효과)판정기준, 평가방법 및 해석방법(통계분석방법)**

**17.1. 유효성 판정기준**

**1) 의료기기 성능평가 전제조건**

(1) 진짜의료기기와 가짜의료기기 식별불가

시험용 의료기기는 외관상 형태, 디자인, 색상 등이 동일하여 육안으로 진짜 시험용 의 료기기와 가짜 시험용 의료기기의 식별이 불가능 하다.

(2) 임상계획서에 따른 시험용 의료기기의의 사용법 준수

피험자가 시험용의료기기를 1일 3회, 1회에 15분간 착용하여야 하며, 또한 착용일지에 는 착용날짜, 착용시간, 착용횟수를 기록한다.

(3) 다기관 임상실시기관에서 임상계획서에 따른 동일 검사지침 준수

착용하기 전 최초 5가지 안구건조증 항목검사《검사항목:안구 표면질환 지수 (Ocular Surface Disease Index, OSDI), 눈물막파괴시간(Tear film break-up time, BUT), 기초눈물 분비량(쉬르머검사), 각막상피점수(Kera toepitheliopathy score), 인상세포학검사(결막 편평상피화생 정도, 술잔세포밀도)와, 착용 후 2주째, 4주째, 8주째 각각 5가지 항목을 검사기준에 의한 증례기록서를 작성한다.

(4) 양측 눈가림 (full double blind)법에 의한 치료지침 준수

본 임상시험용 의료기기는 치료효과 판정에 영향을 미쳐 정보의 비뚤림 유발을 방지하 기 위하여, 피험자, 임상시험책임자, 임상시험담당자 등 본 임상시험 관계자들이 임 상시험이 완료되어 시험용 의료기기를 분해, 진짜 가짜 의료기기로 판명되기 전 까지 는 시험군 대조군을 알 수 없도록 한다.

**2) 의료기기의 치료효과 평가기준**

안구건조증에 사용되는 임상시험용 의료기기는 임상시험기간 동안 피험자가 착용함에 있어서 시험군과 대조군에 따라 치료효과 차이가 발생하는데, 독립표본의 t 검정의 방 법으로 시험군과 대조군의 평균을 비교하여 이들 두 집단 간의 차이가 통계적으로 유 의한지 비교평가 한다.

**17.2. 유효성 평가방법 및 해석**

**1) 유효성 평가방법**

(1) 설문지를 이용한 안구표면질환지수(OSDI) 검사

피험자가 시험용의료기기를 착용하기 전 최초 검사, 2주, 4주, 8주째 피험자의 증상(5 항목), 기능(4항목), 유발인자(3항목)의 12항목을 0부터 4까지의 점수로 분류하여(증상 이 없을 때를 0, 항상 있을 경우는 4), 0부터 100까지의 점수로 표시 기록서를 근거로 하여 공동연구기관(협연센터:전남대학교병원 안과)에서 시험군 대조군의 변화차이를 통계분석법에 따라 의료기기의 치료효과를 비교 평가한다.

(2**)** 형광검사지를 이용한 눈물막파괴시간(BUT) 검사

피험자가 시험용의료기기를 착용하기 전 최초 검사, 2주, 4주, 8주째 피험자의 눈물막 파괴시간을 형광검사지를 이용하여 측정, 기록된 데이터를 근거로 동연구기관(협연센 터:전남대학교병원 안과)에서 시험군 대조군의 변화차이를 통계분석법에 따라 의료기 기의 치료효과를 비교 평가한다.

(3) 쉬르머검사지(Eagle Vision, Memphis TN, USA)를 이용한 기초눈물분비량 검사

피험자가 시험용의료기기를 착용하기 전 최초 검사, 2주, 4주, 8주째 피험자의 쉬르머 검사지(Eagle Vision, Memphis TN, USA)를 이용한 기초눈물분비량 검사하여, 기록된 데이터를 근거로 동연구기관(협연센터:전남대학교병원 안과)에서 시험군 대조군의 변 화 차이를 통계분석법에 따라 의료기기의 치료효과를 비교 평가한다.

**2) 유효성 평가해석**

(1) 1차변수로 설정한 안구표면질환지수(OSDI),눈물막파괴시간(BUT),기초눈물분비량(쉬르 머검사) 검사결과 SPSS에서 P < 0.05 라면 95% 유의수준에서 시험군과 대조군의 차 이가 있는 것으로 안구건조증 치료효과가 있는 것으로 평가한다.

(2) 1차변수인 안구표면질환지수(OSDI),눈물막파괴시간(BUT),기초눈물분비량(쉬르머검사) 와 2차변수인 각막상피병증 점수, 인상세포학검사(결막편평상피화생정도, 술잔세포밀 도) 검사결과 SPSS에서 P = 0.05 라면 95% 유의수준에서는 안구건조증 치료효과가 없는 것으로 평가한다.

**18. 부작용을 포함한 안전성의 평가기준, 평가방법 및 보고방법**

**18.1. 안전성평가 기준**

**1) 안전성평가 항목 및 방법**

발현 된 이상반응은 자세한 설명과 함께 평가항목으로 나열한다. 이상반응이 발생된 경우 임상시험 완료 후 시험용 의료기기를 분해하여 대조군 처리군으로 분류하며, 임상시험용 의료기기와 인과성이 없는 경우 인과성 없음, 인과성이 있는 경우에는 발현 건수와 모든 합병증 발생빈도를 관찰하여 확실함 (certain)상당히 확실함 (probable․likely)가능성 적음 (unlikely)평가 곤란 (conditional․unclassified) 평가불가(unassessable․unclassifiable)로 분류기록, 평가 한다.

| 순번 | 평가항목 | 평 가 방 법 |
| --- | --- | --- |
| 1 | 눈꺼풀 및 안구주위의 독성반응 | 착용 후 시험군의 합병증 발생빈도를 관찰 |
| 2 | 각결막의 안구표면의 독성반응 | 착용 후 시험군의 합병증 발생빈도를 관찰 |
| 3 | 시력 | 착용 후 시험군의 시력의 변화를 측정 |
| 4 | 안압 | 착용 후 시험군의 안압의 변화를 측정 |
| 5 | 기타 이상반응 | 시험용 의료기기의 진짜 가짜에 따라 구분 |

**2) 이상반응조사**

조사자는 임상시험에 사용되는 의료기기 사용 후 나타나는 이상반응여부를 방문일마다 피험자에게 대한 진찰로서 관찰하며, 임상시험용 의료기기와의 인관관계에 대하여 증례 기록서에 기록한다. 이상반응의 정도는 대조군 처리군으로 분류 경증, 중증도, 중증으로 평가한다.

**3) 유해사례의 치료**

신체 검진을 통하여 피험자를 임상적으로 평가하고 실험실적 검사 및 진단적 검사를 실시하며 필요시 다른 과와 협의한다. 임상시험계획서에 준하여 또는 표준적인 치료를 시행하고, 유해사례가 안정되거나 소실될 때까지 추적 관찰한다.

**19. 피험자 동의 설명문 및 동의서 양식**

**피험자를 위한 설명문**

1. 연구제목

□ 안구건조증 임상시험용 의료기기의 안전성 및 유효성 평가

2. 책임연구자

□ 전남대학교 병원 안과 : 윤 경 철

본 설명문 내용에 이해하기 어려운 용어가 포함 될 수 있습니다. 이해가 되지 않는 용 어와 내용은 임상연구담당의사 또는 담당자에게 설명을 요청하십시오.

3. 임상연구의 목적 및 배경

□ 현재 까지 안구건조증에 대한 보편적인 치료방법으로 인공 눈물 점안제를 직접적으로 안구에 투여하였습니다. 하지만 이러한 인공눈물 점안제 직접투여는 면역력 약화와 안구에 직접적으로 투여되여 충혈과 같은 순응도가 떨어지고 여러 가지 부작용을 가지고 있어 이를 피할 수 있는 방법이 요구되어 왔습니다. 최근에는 다양한 치료로 치료효과를 기대하며, 부작용을 최소화한 치료 방법들이 각광을 받고 있는데, 이에 따라 안구건조증 에 대한 저부작용 치료법에 이목이 집중되고 있습니다.

본 임상 시험은 안구건조증을 가지고 있는 환자를 대상으로 안구 비접촉식 안구건조증 치료용 의료기기를 이용한 치료를 시행하여 안구건조증 치료 효과인 안정성 및 유효성을 평가하고자 합니다.

4. 임상시험에 사용되는 의료기기에 관한 정보

□ 본 시험에 사용되는 제품은 의료용자기발생기로 안구비접촉식 안구건조증 치료용 의료 기기입니다.

□ 임상시험용 의료기기는 안면부에 밀착하여 1회에 15분, 오전 오후 저녁 1일 3회 착용하며, 착용 후에는 1분간 상하좌우 눈 운동을 한다.

5. 임상연구절차 및 검사

□ 본 연구는 피험자 모집일로부터 4개월 동안 진행되며 동의서를 작성 후 약 4회 병원에 방문하여야 합니다. 각 방문 시 마다 시험책임자로부터 적절한 상담을 받으시게 됩니다.

□ 연구시작시 인구학적 검사, 병력 및 과거력조사, 기타 조사를 실시하며, 피험자로서 적 합한지 판단합니다. 이러한 선정과정을 거쳐 임상연구에 적합한 것으로 판단되고, 귀하 가 임상연구에 참여함을 서면으로 동의 하실 경우, 임상시험에 참여하실 수 있습니다.

□ 위 모든 내용은 증례 기록서에 기록하게 되며, 총 연구기간 동안 피험자, 보호자께서 궁금하신 임상시험 절차에 대해서는 ‘언제든지’ 설명해 드리며, 시험담당자가 직접 개별적으로 전화를 드려 임상시험에 이상반응 유. 무를 확인 할 수 도 있습니다.

6. 피험자가 준수하여 할 사항

□ 귀하는 임상시험 제반사항을 충분히 숙지하고, 관련 규정에 따라 성실히 임상시험을 수행해야 합니다.

□ 시험용 의료기기 사용과 병용금기치료

임상시험기간 동안 피험자는 약물복용을 금기하며 또한 본 임상시험용 의료기기이외에 다른 안구건조증 치료관련 행위를 할 수 없다. 시험용 의료기기를 사용하는 임상기간 동안 안구건조증 질환이 악화되거나 다른 질환으로 피험자가 치료를 호소한 경우 예외 적으로 IRB 위원회에 보고 후 피험자 지위에서 탈락되며, 질환에 따르는 약물을 사용한 다.

□ 임상시험용 의료기기 사용시 주의사항

피험자는 임상시험기간 동안 임상시험용 의료기기를 사용하기 전에는 다음의 사항을 주 의하여야 한다.

1) 시계, 전기카드 등의 전기의 영향을 받는 물건에는 임상시험용 의료기기를 가까이 대지 않는다.

2) 임상시험용 의료기기의 자석 장착부에 파손이 있을 시에는 사용을 중지한다.

3) 임상시험용 의료기기의 표면에 금속 등의 이물이 흡인, 부착되지 않았음을 확인하여야 한다.

4) 임상시험용 의료기기 사용시 손상이 발견된 경우에는 사용을 즉시 중지한다.

5) 신체에 이상을 느낀 경우에는 사용을 즉시 중지한다.

6) 임상시험용 의료기기 착용으로 닿는 안면부에 발진, 발적, 가려움 등의 증상이 나타난 경우에는 사용을 즉시 중지한다.

7) 임상시험용 의료기기는 피험자가 개조하지 않는다.

8) 임상시험용 의료기기의 파손으로 인한 부속품은 지정된 것을 사용하여야 한다.

9) 임상시험용 의료기기는 내 측면 다리에 표시된 Eye Plus Alpha는 제품의 명칭이며, 전면에 표시된 “임상시험용”이라는 글씨는 본 제품의 임상시험용이라는 것을 뜻한다.

□ 임상시험 중지․탈락 기준

1) 중지 기준

○ 임상시험의뢰자는 임상시험용 의료기기의 안전성 등의 사유로 임상시험을 중지하고자 할 경우 임상시험심사위원회에 임상시험중지 요청을 하여 임상시험위원회의 결정에 따 라 임상시험을 중지 할 수 있다.

○ 발생된 이상반응 처치를 위하여 일시적으로 중지되는 경우.

○ 심각한 이상반응/이상의료기기반응의 발생으로 중지되는 경우.

2) 중지의 처리

○ 임상시험이 중지 된 경우 임상시험책임자는 중지 된 시점까지 진행된 피험자에 대한 증례기록서, 임상시험 진행현황 및 결과를 정리하여 임상시험 의뢰자에게 전달하며 모 든 시험 관련자료(증례기록서 및 임상시험의료기기)를 임상시험의뢰자에게 반납하여야 한다.

○ 임상시험이 중지된 경우 임상시험책임자는 시험 중지 사실과 함께 IRB에 서면으로 통 보하여야 한다.

3) 탈락 기준

○ 피험자 또는 법적 대리권자가 임상시험 참여 중단을 요청한 경우

○ 안전성, 유효성 평가에 영향을 줄 수 있는 수술, 약물 또는 다른 의료기기를 병행하여 사용한 경우

○ 심각한 부작용이 발생한 경우

○ 치료방법을 제대로 수행하지 않는 경우

○ 피험자가 시험자의 지시에 불응하거나 또는 동의서에 제시된 사항을 준수 하지 않아 그 유효율의 평가에 영향을 미치는 경우

○ 임상시험과 관련이 없는 사유로 피험자가 사망한 경우

○ 기타 임상시험담당자가 임상시험 진행에 문제가 있다고 판단되는 경우

4) 탈락의 처리

○ 피험자가 중도 탈락 된 경우 탈락사유 및 탈락 전까지 진행된 임상시험 관련 자료를 기록, 보관한다.

○ 시험도중 피험자가 내원하지 못한 경우 피험자간의 건재 여부를 확인하고 그 이유를 분명하게 하여야 한다.

○ 중도 탈락된 자는 타당한 이유 또는 근거가 없는 한 안전성. 유효성평가 통계처리에 포함 된다.

7. 임상연구에 수반될 수 있는 위험

□ 임상연구 중 안전성에 문제가 있어 위험하다고 판단 될 경우 귀하의 임상연구 참여가 중단 될 수 있습니다.

8. 임상시험 도중 중지된 경우

□ 귀하가 본 임상연구에 참여하기를 원하지 않는다면, 귀하의 임상시험책임자는 귀하에 게 안구 약제의 복용이나 점안제 등의 치료에 대해 설명 할 수 있으며, 원하는 방법으 로 치료를 받으실 수 있습니다.

9. 예상되는 이점

□ 본 임상연구에 참여함으로써 귀하에게 의학적 혜택이 보장되는 것은 아닙니다. 그러나 이 연구에서 얻은 정보는 유사한 질환을 가진 환자의 더 나은 치료에 도움이 될 수 있 습니다.

10. 자발적 참여

□ 본 임상연구의 참여는 귀하의 자발적 참여의사에 의해 결정됩니다. 귀하는 참여하지 않을 권리가 있으며, 시험을 그만 둘 수 있습니다. 귀하가 본 연구에 참여하지 않아도 아무런 불이익을 받지 않으며, 귀하의 결정을 향 후 진료를 받는 것에 영향을 미치지 않으며, 참여를 결정한 경우에도 어떠한 편견, 불리함 또는 본 연구기관에서의 이득 손 실없이 언제라도 임상연구의 참여를 중단 할 수 있습니다.

11. 임상시험과 관련 된 손상이 발생하였을 경우 피험자를 대상으로 보상이나 치료방법

□ 본 임상시험 기간 동안 의료기기 또는 임상시험 절차사의 문제로 인하여 피험자에게 이상반응이 발생한 경우 임상시험책임자에게 알려야하며, 적절한 치료를 무상으로 받 을 수 있습니다.

12. 피험자가 임상시험에 참여함으로 써 받게 될 금전적 보상

□ 귀하는 임상시험에 참가하는 것에 대한 금전적 보상을 받지 않습니다. 하지만 귀하께 서 임상시험을 위한 임상시험 실시기관에 총 4회 방문하여 방문시 소요되는 교통비(3만 원 X 4회방문=120,000원)는 임상시험 종료 후 일괄적으로 지급됩니다. 또한 임상시험 기간 동안 피검자의 검사에 소요되는 검사비용(100,000원)은 임상시험기관에서 부담합니 다.

13. 임상시험에 참여함으로써 피험자에게 발생 할 수 있는 예상 비용에 관한 사항

□ “피험자 보상에 관한 규약”에 따릅니다.

14. 비밀보장

□ 본 임상연구와 관련되는 모든 기록을 엄격하게 관리함으로서 피험자의 비밀이 보장되 며 , 임상시험결과의 발표 또는 출판 시에는 모든 피험자의 자료가 익명으로 다루어지 며, 사진이 실리는 경우에는 신원을 확인할 수 없도록 편집되어 집니다. 다만 식품의 약품 안전청 또는 임상시험심사위원회의 요구가 있을 때에는 피험자의 기록이 제공 될 수 있습니다.

□ 임상시험의 일부로 여러분의 개인기록, 의료기록, 검사결과, 건강정보 등이 수집되게 됩니다. 이러한 정보는 임상시험 담당의사, 연구담당, 직원이 이러한 정보를 사용할 수 있으며 임상시험의 절차와 자료의 신뢰성을 검증하기 위해서 본 연구를 감독하는 정부 기관과 병원 내 임상시험심사위원회도 여러분이 동의를 한다는 것은 이러한 정보의 사 용을 허락한다는 것입니다.

15. 임상시험참여가 중지 되는 경우 및 해당사유

□ 임상 시험 진행 중 관찰되는 상황이 임상시험을 계속 진행하는 것에 무리가 있다고 판

단 되는 경우에는 임상시험책임자는 임상시험심사위원회에 임상시험 중지요청을 하여

야하고, 임상시험심사위원회의 결정에 따라 임상시험을 중지 할 수 있습니다.

16. 기타

□ 귀하는 본 임상시험에 참여함으로서 임상시험과 관련 된 어떤 의문점이라도 질문을 하여 의료진으로부터 이에 대한 충분한 설명을 들을 권리가 있으며, 임상연구 중에 귀 하의 임상시험 지속 참여의지에 영향을 줄 수 있는 어떠한 새로운 정보라도 본 의료진 은 즉시, 본인 또는 대리인에게 상기 정보에 대한 내용을 알려 드리겠습니다.

□ 만일 귀하께서 이 연구에 피험자로서 귀하의 권리에 관하여 문의하실 것이 있거나 또 는 연구진행 중 어떠한 문제가 발생하였을 경우, 추가적인 정보를 얻고자 할 경우에는 임상시험 책임자(김재찬 02-748-9838)로 연락하여 주십시오.

□ 피험자의 권리에 대하여 의문이 있는 경우, 전남대학교병원 생명의학연구윤리심의위원

회 (062-220-5257)로 연락하실 수 있습니다.

□ 피험자는 시험담당자에게 피험자 설명문 및 동의서의 사본을 받을 수 있습니다.

**피 험 자 동 의 서**

연구 제목 : 안구건조증 임상시험용 의료기기의 안전성 및 유효성 평가하기 위한 임상시험

1. 본인은 본 임상시험의 목적 및 방법, 임상연구의료기기의 기대효과, 가능한 위험성, 타 치료방법의 유ㆍ무 등에 대하여 충분한 설명을 듣고 이해하였습니다.

2. 본인은 위험과 이득에 관하여 들었으며 나의 질문에 만족할 만한 답변을 얻었습니다.

3. 본인은 이후의 치료에 영향을 받지 않고 언제든지 연구의 참여를 거부하거나 연구의 참

여를 중도에 철회할 수 있고 이러한 결정이 나에게 어떠한 해가 되지 않을 것이라는 것 을 알고 있습니다.

4. 본인은 본인에 대한 모든 자료에 대하여 비밀이 보장됨을 알고 있습니다.

5. 본인은 이 동의서의 사본을 받을 것을 알고 있습니다.

6. 본인은 이 연구에 참여하는 것에 대하여 자발적으로 동의합니다.

7. 본인이 만20세 미만의 미성년자인 경우에는 본인의 승낙과 법정대리인의 동의가 필요합니다.

피험자 성명 서명 날짜 / /

대리인 성명 서명 날짜 / /

담당연구원 성명 서명 날짜 / /

담당책임자 성명 서명 날짜 / /

**20. 피해자 보상에 대한 규약**

**20.1. BM생명공학연구소(주)는**

의료기기 임상시험관리기준에 따라 이루어진 임상시험에 있어서 임상시험용 의료기기 로 인하여 피험자에게 발생한 유해하고 의도되지 않은 반응에 의한 피험자의 신체상 의 손상에 대하여, 다음의 경우에 본 보상규약 및 관련 법률의 규정에 따라 이를 보상 합니다.

**20.2. 보상요건**

본 보상규약에 따른 피험자 보상은 다음의 요건 하에 이루어집니다.

1) 본 임상시험용 의료기기로 인하여 발생한 신체상의 손상일 것.

2) 시험자가 식품의약품안전청장의 승인을 받은 임상시험계획서의 제반 내용을 준수하였을 것

3) 시험자의 명백한 과실이나 의무태만에 기인하지 아니하였을 것

4) 피험자가 시험책임자 또는 시험담당자의 제반 지시사항을 모두 준수 하였을 것

5) 피험자가 당해 신체상의 손상으로 인한 손해의 발생을 최소화하기위하여 조치를 하였 을 것

**20.3. 보상 제외사유**

1) 임상시험용 의료기기로부터 기대 된 효과, 효능의 불충분으로 의한 손상

2) 피험자의 부주의로 인하여 발생한 손상

**20.4. 보상기준**

1) 예상 된 의료기기 이상반응에 대하여 당사자들 간에 미리 합의한 보상액 또는 조치 가 있는 경우, 당해 기준에 따라 이를 보상합니다.

2) 그 외에 경우에는 신체손상의 정도, 성격, 지속기간, 유사사례 등을 종합적으로 고려 하여 당사자들 간에 합의한 보상방법에 따라 이를 보상합니다.

3) 당사자들 간에 전항의 합의가 이루어지지 아니한 경우에는, 법원의 판결 및 이에 준

하는 결정의 확정내용에 따라 보상합니다.

**20.5. 보상절차**

1) 본 보상규약에 따른 신체상의 손상을 입은 피험자는 임상시험의 시험책임자나 시험 기관에 먼저 필요한 의료조치를 요청하여야 합니다.

2) 시험책임자나 시험기관의 조치에도 불구하고 신체상의 손상이 완치되지 아니한 피험 자는 의뢰기관에 대하여 이에 대한 보상을 요청할 수 있습니다.

3) 의뢰기관은 위 보상요청을 받은 후 지체 없이 보상대상 해당여부 및 보상기준에 대 한 조사를 마치고 이에 관한 내용을 피험자에게 통보하여야합니다.

4) 피험자는 위 통보내용에 의의가 있는 경우, 위 통보를 받은 날로부터 영업일(5) 이내 에 이에 대한 이의내용을 의뢰기관에 통보하여야 합니다.

5) 피험자가 제(3)항의 통보를 받고도 이에 대한 이의를 통보하지 아니한 경우, 양 당사 자는 위 통보내용에 따른 보상에 합의한 것으로 양해합니다.

6) 피험자가 제(4)항의 규정에 따라 이의를 통보한 경우, 의뢰기관은 피험자에게 위 보 상 대상 해당여부 및 보상기준에 관하여 판단할 객관적인 전문가를 복수로 추천하고, 피험자를 위 추천일로부터 영업일(3)내에 추천인 1명을 지명합니다. (피험자가 지명하 지 않을 경우 의뢰자가 임의 택일합니다)

**20.6. 적용범위**

1) 본 보상규약은 의뢰기관이 의뢰하는 모든 임상시험에 참여하는 피험자에 의뢰기관과 피험자 간에 다른 약정이 없는 한 그 범위 내에서 일반적으로 적용됩니다.

2) 피험자가 임상시험에 관한 보상에 대하여 의뢰기관의 승인을 받지 아니하고 임상시험 과 관련된 다른 제3자와 체결한 일체의 합의내용은 의뢰기관에 대하여 효력이 없습니 다.

폐사는 위의 여러 제반 내용을 참고하여 피험자가 본 임상시험에 의해 어떠한 불이익 이라도 받지 않도록 주의하며, 만약 본 임상시험에 의해 문제점이 발생한 경우 피해자 보상 규약에 의거하여 책임질 것을 서약합니다.

2012년 10월 일

의뢰자 : BM생명공학연구소(주)

직 명 : 대표이사 성 명 : 김 희 구 (인)

|  | | | | **증례기록지**  **(CRF: Case Report Form)**  **안구건조증 임상시험용 의료기기의 안전성 및 유효성을 평가하기 위한 임상시험**  **시험기간 : IRB 승인일로부터 4개월 간**  **피시험자 배정번호 :**  **이 름(이니셜) :**  **성별 및 나이 : (남 / 여), 만세**  **연락처: ( ) -**  **연구책임자 윤 경 철**  **전남대학교 의과대학 안과학교실**  **의뢰기관 : 전남대학교 의과대학 안과학교실**  **Work Sheet 년 월 일** | | | | | | | | | | | | | | | | | | | | | | | |  | | | |
| --- | --- | --- | --- | --- | --- | --- | --- | --- | --- | --- | --- | --- | --- | --- | --- | --- | --- | --- | --- | --- | --- | --- | --- | --- | --- | --- | --- | --- | --- | --- | --- |
|  | | | |  | | |  | | | | | |  | | | | |  | | | | | | | | | |  | | | |
| **피험자 Intials** | |  | | | | | **시험용 의료기기번호** | | | | | |  | | | | | **피험자일련번호** | | | | | | |  | | | | | | |
|  | | | |  | | |  | | | | | |  | | | | |  | | | | | | | | | |  | | | |
| **1. 시력/병력** | | | |  | | |  | | | | | |  | | | | |  | | | | | | | | | |  | | | |
|  | | | |  | | |  | | | | | |  | | | | |  | | | | | | | | | |  | | | |
| **시력** | **우안: ( x Dsph= Dcyl x )** | | | | | | | | | | | | | | | | | | | | | | | | | | | | | | |
|  | **좌안: ( x Dsph= Dcyl x )** | | | | | | | | | | | | | | | | | | | | | | | | | | | | | | |
| **안압** | **우안: mmHg / 좌안: mmHg** | | | | | | | | | | | | | | | | | | | | | | | | | | | | | | |
|  | | | |  | | |  | | | | | |  | | | | |  | | | | | | | | | |  | | | |
| **2. 증상소견항목평가** | | | | | | **□좌안 □우안** | | | | | | | | | | | | | | | | | | | | | | | | | |
|  | | | |  | | |  | | | | | |  | | | | |  | | | | | | | | | |  | | | |
| **증상** | **눈꺼풀** | | | | | **□없음** | | | | **□통증** | | | | | | **□소양감** | | | | | | **□발적** | | | | | | | | **□기타:** | |
|  | **안구표면** | | | | | **□없음** | | | | **□충혈** | | | | | | **□상피손상** | | | | | | **□눈물** | | | | | | | | **□기타:** | |
| **소견** | **각막상피병증** | | | | | **□Grade 0** | | | **□Grade I** | | | | | **□Grade II** | | | | | | **□Grade III** | | | | **□Grade IV** | | | | | | | **□Grade V** |
|  | **TBUT** | | | | | **□Variable( 초)** | | | | | **□6-10초( 초)** | | | | | | | **□1-5초( 초)** | | | | | **□Immediate** | | | | | | | | |
|  | **Schirmer** | | | | | **□Variable( mm)** | | | | | | **□5＜∼≦10mm( mm)** | | | | | | | **□2＜∼≦5mm( mm)** | | | | | | | | **□0＜∼≦2mm( mm)** | | | | |
|  | **편평상피화생** | | | | | **□Grade 0** | | | | | | **□Grade 1** | | | | | | | **□Grade 2** | | | | | | | | **□Grade 3** | | | | |
|  | **술잔세포밀도** | | | | |  | | | | | | | | | | | | | | | | | | | | | | | | | |
|  | **결막충혈** | | | | | **□없거나 가벼운 충혈** | | | | | | | | | | | | **□심한 충혈** | | | | | | | | | | | | | |
|  | **눈꺼풀** | | | | | **□안건염** | | | | | | | | | | | | **□Trichiasis, Keratinization, Symblepharon** | | | | | | | | | | | | | |
|  | **눈물층** | | | | | **□경한 Debris / Tear meniscus 감소** | | | | | | | | | | | | **□심한 Debris / Mucus clumping** | | | | | | | | | | | | | |
|  | | | |  | | |  | | | | | |  | | | | |  | | | | | | | | | |  | | | |
| **진단분류** | | | | | **□건성안 의증** | | | **□LevelⅠ** | | | | | | | **□LevelⅡ** | | | | | | **□LevelⅢ** | | | | | | | | **□LevelⅣ** | | |
|  | | | |  | | |  | | | | | |  | | | | |  | | | | | | | | | |  | | | |
| **3. 시험검사** | | | |  | | |  | | | | | |  | | | | |  | | | | | | | | | |  | | | |
|  | | | |  | | |  | | | | | |  | | | | |  | | | | | | | | | |  | | | |
| **◎ 임상시험검사 시험때 내용을 기록하여 주십시오** | | | | | | | | | | | | | | | | | |  | | | | | | | | | |  | | | |
| **처방** | **치료지침** | | | | | | | | | | | | | | | | **약제명** | | | | | | | | | **용법/용량** | | | | | |
| **□** | 환자교육, 환경조절 | | | | | | | | | | | | | | | |  | | | | | | | | |  | | | | | |
| **□** | 인공눈물(□방부제 □무방부제) □HA □CMC □기타 | | | | | | | | | | | | | | | |  | | | | | | | | |  | | | | | |
| **□** | 젤, 연고 | | | | | | | | | | | | | | | |  | | | | | | | | |  | | | | | |
| **□** | 0.05% topical cyclosporine | | | | | | | | | | | | | | | |  | | | | | | | | |  | | | | | |
| **□** | Topical steroid | | | | | | | | | | | | | | | |  | | | | | | | | |  | | | | | |
| **□** | Oral tetracyclines | | | | | | | | | | | | | | | |  | | | | | | | | |  | | | | | |
| **□** | Oral essential fatty acid | | | | | | | | | | | | | | | |  | | | | | | | | |  | | | | | |
| **□** | punctual plug/occlusion □plugs(제품명: ) □cautery □surgery | | | | | | | | | | | | | | | | | | | | | | | | | | | | | | |
| **□** | 기타: | | | | | | | | | | | | | | | |  | | | | | | | | |  | | | | | |
| **□** | 기타: | | | | | | | | | | | | | | | |  | | | | | | | | |  | | | | | |
| **◎ 기존 처방의 변경 / 추가시 반드시 사유를 기록하여 주십시오** | | | | | | | | | | | | | | | | | | | | | | | | | | | | | | | |
| □약제부작용 | | | □충혈 □분비물 □가려움증 □자극감 □기타( ) | | | | | | | | | | | | | | | | | | | | | | | 해당약제( ) | | | | | |
| □효과물충분 (약제의 치료효과가 불충분하거나 환자 순응도가 낮은 경우 등) | | | | | | | | | | | | | | | | | | | | | | | | | | | | | | | |
| **기타** |  | | | | | | | | | | | | | | | | | | | | | | | | | | | | | | |
|  | | | |  | | |  | | | | | |  | | | | |  | | | | | | | | | |  | | | |
| **4. 종합평가 환자의 상태를 전체적으로 평가한다면? (해당 사항에 (v)로 표기)** | | | | | | | | | | | | | | | | | | | | | | | | | | | | | | | |
|  | | | | | | | | | | | | | | | | | | | | | | | | | | | | | | | |
| **□ 매우나쁨 □ 나쁨 □ 보통 □ 좋음 □ 매우좋음** | | | | | | | | | | | | | | | | | | | | | | | | | | | | | | | |

**의사명 서명**

| **회차** | | **건성안 설문지** | | | | | | | | | | | | | | **년 월 일** | | | |
| --- | --- | --- | --- | --- | --- | --- | --- | --- | --- | --- | --- | --- | --- | --- | --- | --- | --- | --- | --- |
|  | |  |  |  | | | |  | | | | | | | |  | | | |
| **피험자 Intials** |  | | **시험용 의료기기번호** |  | | | | **피험자일련번호** | | | | |  | | | | | | |
| 본 설문지는 안구건조증 치료용 의료기기의 안전성 유효성 평가를 위해 보다 효과적이고 적합한,  안구건조증 치료용 의료기기의 효능 효과를 검증하는데 소중한 자료로 사용하고자 합니다.  본 설문에 포함된 내용은 건성안 치료가이드라인 개발자료 이외의 목적에는 사용하지 않습니다.  다음 각각의 설문항목 중 해당되는 내용에 표시해 주십시오. | | | | | | | | | | | | | | | | | | | |
|  |  | |  |  | | | |  | | | | |  | | | | | | |
| **1. 지난 한 주 동안 아래의 증상을 얼마나 경험하셨습니까?** | | | | | | | | | | | | | | | | | | | |
|  | | | | | | | | | | | | | | | | | | | |
|  | | | | **항상** | **대부분** | | | | **절반정도** | | | **가끔** | | | | | **전혀** | | |
| **1. 눈부심** | | | | **□** | **□** | | | | **□** | | | **□** | | | | | **□** | | |
| **2. 이물감 (모래알 들어간 느낌)** | | | | **□** | **□** | | | | **□** | | | **□** | | | | | **□** | | |
| **3. 통증 혹은 시린 느낌** | | | | **□** | **□** | | | | **□** | | | **□** | | | | | **□** | | |
| **4. 번져 보임** | | | | **□** | **□** | | | | **□** | | | **□** | | | | | **□** | | |
| **5. 시력 저하** | | | | **□** | **□** | | | | **□** | | | **□** | | | | | **□** | | |
|  |  | |  |  | | | |  | | | | |  | | | | | | |
|  |  | |  |  | | | |  | | | | |  | | | | | | |
| **2. 지난 한 주 동안 안구건조증상으로 인해 다음의 활동에 지장을 받으셨습니까?** | | | | | | | | | | | | | | | | | | | |
|  | | | | | | | | | | | | | | | | | | | |
|  | | | **해당사항**  **없음** | **항상** | | | **대부분** | | | | **절반정도** | | | | **가끔** | | | | **전혀** |
| **6. 독서나 신문 읽기** | | | **□** | **□** | | | **□** | | | | **□** | | | | **□** | | | | **□** |
| **7. 운전** | | | **□** | **□** | | | **□** | | | | **□** | | | | **□** | | | | **□** |
| **8. 컴퓨터** | | | **□** | **□** | | | **□** | | | | **□** | | | | **□** | | | | **□** |
| **9. TV 시청** | | | **□** | **□** | | | **□** | | | | **□** | | | | **□** | | | | **□** |
|  |  | | *해당사항없음인 경우 우측의 내용은 표기하지 않습니다. | | | | | | | | | | | | | | | | |
|  |  | |  |  | | | |  | | | | |  | | | | | | |
|  |  | |  |  | | | |  | | | | |  | | | | | | |
| **3. 지난 한 주 동안 아래 상황에서 눈이 불편한 적이 있습니까?** | | | | | | | | | | | | | | | | | | | |
|  | | | | | | | | | | | | | | | | | | | |
|  | | | **해당사항**  **없음** | **항상** | | **대부분** | | | | **절반정도** | | | | **가끔** | | | | **전혀** | |
| **10. 바람부는 실외** | | | **□** | **□** | | **□** | | | | **□** | | | | **□** | | | | **□** | |
| **11. 건조한 실내 (백화점, 은행)** | | | **□** | **□** | | **□** | | | | **□** | | | | **□** | | | | **□** | |
| **12. 에어컨/히터가 있는 밀폐되 공간** | | | **□** | **□** | | **□** | | | | **□** | | | | **□** | | | | **□** | |
|  | | | *해당사항없음인 경우 우측의 내용은 표기하지 않습니다. | | | | | | | | | | | | | | | | |
|  |  | |  |  | |  | | | |  | | | |  | | | |  | |
|  |  | |  |  | | | |  | | | | |  | | | | | | |
| **4. 지난 한 주 동안의 안구건조에 대한 증상을 종합적으로 평가한다면? (해당 사항에 (V)로 표기)** | | | | | | | | | | | | | | | | | | | |
|  |  | |  |  | | | |  | | | | |  | | | | | | |
| **□ 매우나쁨 □ 나쁨 □ 보통 □ 좋음 □ 매우좋음** | | | | | | | | | | | | | | | | | | | |
|  |  | | | | | | | | | | | |  | | | | | | |
| 본 설문에 참여하여 성심껏 답변해 주셔서 감사합니다. | | | | | | | | | | | | | | | | | | | |
|  | | | | | | | | | | | | | | | | | | | |
